# The effect of control rods on the reactivity and flux distribution of BWR 4 bundle using MCNPX Code

**DOI:** 10.1038/s41598-021-88067-0

**Published:** 2021-04-22

**Authors:** Sayed. Saeed. Mustafa

**Affiliations:** Faculty of Science, Zagazig University, Zagazig, Egypt

**Keywords:** Nuclear physics, Theoretical nuclear physics

## Abstract

This paper has three main objectives related to the neutronic and burnup analysis of the BWR (Boiling Water Reactor) Four-Lattice. The first objective is to provide partial validation of the MCNPX code for this lattice by comparing its results with Scale-5.1 results. Validation of the MCNPX to calculate effective multiplication factor and reactivity rod worth for the F-Lattice is provided. This is carried out in case of instantly removing the control blade and replacing it with a graphite moderator. Moreover, spatial neutron flux distributions using F-mesh card over the bundle and the control blade are investigated at inserting and withdrawing the B_4_C. The second objective is to perform parametric design studies of the F-Lattice. Areas of particular interest are the effect of increased or decreased blade width on the neutron flux throughout the bundle. It is found that the presence of carbon in the control blade at withdrawing the B_4_C makes the reactor supercritical, (K-eff = 1.22206). On the other hand, the use of B_4_C blade presents (K-eff = 0.93521). Consequently, the reactivity of 10% B_4_C thinner case is higher that of 10% B_4_C thicker. The simulation also showed that the B_4_C blade had an effective role in decreasing the thermal flux at the periphery of the bundle. This is contrast to the effect of carbon that moderates fast to thermal neutrons. The third part of this work aims at studying the burnup calculations using MCNPX code for 30 days burn with 1 day time step then for 20 months burn with 2 week time steps for the lattice. At the end of the work, it is very important to determine the most proper bundle model that achieves a prolonged fuel burn and flatting thermal flux distribution. For reaching this goal, three cases (B_4_C, 10% thinner of B_4_C and 10% thicker) are simulated by MCNPX code till 70 GWd/ton. It is found that the B_4_C and 10% thicker are the appropriate models that can satisfy the safety considerations of the Compact Modular Boiling Water Reactor.

## Introduction

The Four-Bundle Lattice, known as the F-Lattice, is an innovative design that reduces the total number of control blades employed in the Boiling Water Reactor (BWR) design. This Lattice type utilizes a staggered row configuration for the control blades. These control blades are characterized by their large width. This large width allows each control blade to cover two bundles in each direction, resulting in four bundles, or one assembly, to be entirely enclosed by two control blades; thus it is named after Four-Bundle Lattice.The Compact Modular Boiling Water Reactor (CM-BWR) is one the reactors that are considered the main interests of the nuclear power industries. It is designed to produce 350 MWe (electric power). The modeling of such reactor depends on repeating the unique F-lattice. The F-Lattice is characterized by a staggered row configuration for the control blades. These control blades are wider in thickness than those used in the most commercial boiling water reactors nowadays. The increase in blade width and pitch allows for approximately a one-half factor decrease in the number of control blades. The reduction in the number of control blades results in minimizing the construction and maintenance costs, as well as reducing the risk of failures within the core^1^.

The safety and cost are two important factors required for the development of new products in the nuclear power plants. Therefore, any possible modification to a nuclear reactor that can decrease cost and improve or maintain safety is considered highly desirable. Many new reactor designs that achieve these goals have been proposed. The Compact Modular Boiling Water Reactor (CM-BWR) is one of these reactor designs. The main goal of designing such nuclear reactors is to minimize the complexity, size, cost and number of overall parts in the reactor^2^.

When F-Lattice is utilized in the ESBWR (Economically Simplified Boiling Water Reactor) design, only half the number of control rods is required in the core. Fewer control rods means a reduction in material and manufacturing costs, fewer drives and hydraulic control units. Furthermore, there would have been approximately half the number of control rod related components requiring maintenance or posing the risk of failure. This is accompanied by a reduction in cost and an increase in safety; the two key goals of all design modifications in a nuclear reactor. While the F-Lattice is no longer a part of the ESBWR, it still remains a key component for the CM-BWR design. Therefore, study and analysis of this new lattice design must be performed before the CM-BWR design can be completed^1,3^.

Generally, the production of electricity by boiling water reactors is one of the basic considerations of power plants because of reactor power raising and higher fuel assembly burnups. Highly reliable thermal margins and stability assessment are required for BWRs operating at higher powers. Higher burnup required higher average U-235 enrichment in the fresh assemblies and led to more heterogeneous cores, which stressed the thermal and shutdown margins of the reactor. The required better thermal and shutdown margins were gained by significantly improving BWR fuel assembly designs^4^.

The fuel characteristics of BWRs are studied less than PWRs. However, BWRs are widely deployed internationally. This is attributed to the complexity of the modeling associated with their highly heterogeneous fuel assemblies^5–7^. This has been recently confirmed for an advanced BWR with a published core design which had been modeled by experts in reactor physics^8^.

The infinite multiplication factor for the F-lattice is evaluated when the thickness of B_4_C blade is increased and decreased by 10% of its nominal thickness. It is expected that the decreased thickness of B_4_C leads to an increase in the effective multiplication factor due to the low boron content in this case.

The reactivity worth of control rod is another important safety parameter that is affected by the variation of isotopes during fuel burnup and the operational time of the rod and its position in the core^9^. This parameter can be calculated using the equation ρ = $\frac{{K - K_{0} }}{K}$, where $K_{0}$ is the multiplication factor with control rods inserted in, and $K$ is the multiplication factor with the control rods pulled out and replaced with pure graphite. This means that when B_4_C is replaced by graphite, neutron absorption dramatically decreases, causing an increase in neutron population, increase in flux and a larger effective multiplication factor. All the obtained results are accompanied by calculating the standard deviation and the percent error relative to Scale-5.1 code.

Because of the strong dependence of reactor safety on the flux distribution, F-Mesh card is used for plotting the thermal, epithermal, fast and total flux distributions in xy-plane of the F-bundle. To accomplish this, the three dimensions of the assembly geometry are divided into 10 meshes. Spatial flux distribution is not only studied for the fuel F-bundle, but also plotted over the control blade at inserting and withdrawing the B_4_C. This is conducted for distinguishing between the role of both B_4_C as a strong absorber and graphite as a strong moderator in determining the flux profile.

The last section of this work, depletion calculations of BWR bundle, focused mainly on searching for which model among the three models under investigation (B_4_C(nominal case), 10% B_4_C thinner and 10% B_4_C thicker) can achieve a prolonged life cycle and flattened thermal flux distribution. These two features are one of the desired safety considerations of the BWR cores for enhancing the stabilization of reactor operation. As we will see, the B_4_C and 10% B_4_C thicker can satisfy these goals.

## Methodology

The MCNPX code was developed by the Los Alamos National Laboratory. It is a general purpose Monte Carlo code, which facilitates independent or coupled neutron, photon and electron transport calculations^10^. All the neutronic and burnup calculations in this research are conducted by this code. It is capable of modeling complex 3D geometries and utilizes extensive point-wise cross-section data library on a continuous energy spectrum. The cross section data library used in the calculations is the updated library, endf71X. This data library provides the necessary probability distributions for simulating particle interactions through use of random number sampling^11^. Through a large number of neutrons and active cycles, MCNPX is capable of determining k-effective, neutron flux, current and many other neutronic parameters. Increasing the number of histories and the number of neutrons tracked in each history provides more detailed results, though at the expense of higher computational costs.

Flux calculations require the use of a mesh, which divides the model into different cells. Each individual cell describes a grouping of fuel, gap, clad and surrounding water as one geometric unit. Flux results are then averaged within each geometric unit. A more refined mesh leads to smaller cells, which reduces the effect of averaging. Smaller cells lead to more accurate flux profiles. The flux, or power, distribution can be determined with the average flux results for each cell. The superimposed mesh that covers only desired specific locations in the model can be specified in MCNP6 which is involved in MCNPX code. The FMESH tally is a general track-length tally which does not record tracks in pre-assigned geometry cells, but uses a mesh superimposed over the problem geometry^12,13^. The mesh can be defined in Cartesian or cylindrical coordinates (Supplementary Material).

## The BWR bundle under investigation

**Figure 1:**
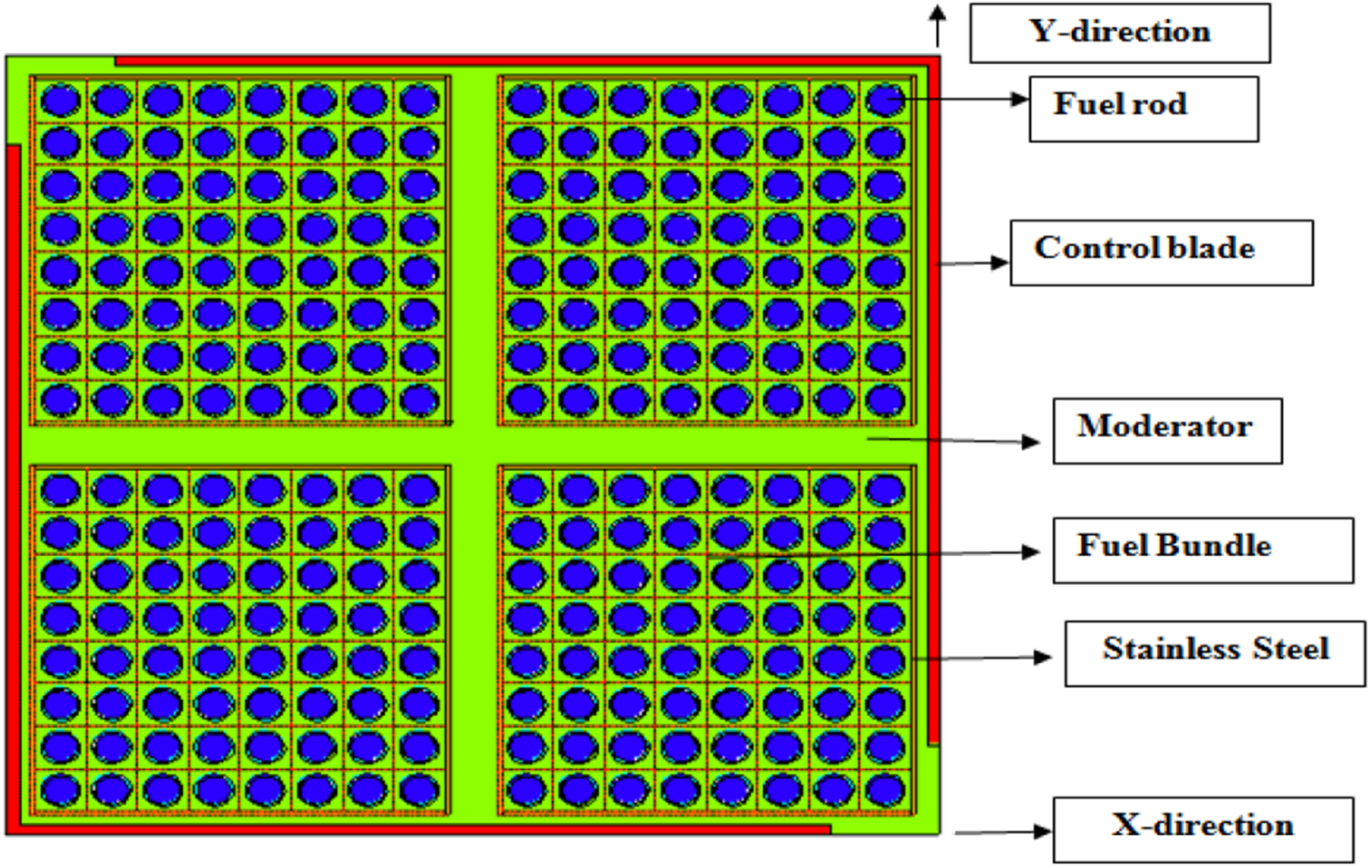
4-bundle cell F-Lattice core configuration used for analysis.

The F-lattice was modeled by MCNPX code as a collection of unit cells. A unit fuel cell is composed of the fuel, gap, clad and surrounding moderator. This unit fuel cell was then repeated using the MCNPX “lattice fill” option to create an 8 × 8 unit bundle. A SS-304 canister was then modeled around the unit bundle. This unit bundle was then repeated four times with moderator spacing between them. This was done using the “like m but” command in MCNPX. This command allows a unit to be repeated with slight changes in material or physical location. The control rods were then modeled around the four bundles. This completed the 4-bundle cell. The parameters and dimensions of F-bundle are depicted in Table 1. A schematic diagram of F-lattice is given in Fig. 1. The source used was the “ksrc”, which allows the user to define specific locations for the initial sources to be located. Sources were placed in the center of several different fuel rods throughout the system. MCNP6 automatically expands its source locations with each generation history, using information from each previous history to better approximate the next. Finally, the F4 tally was utilized for the flux analysis. Neutron energy levels can also be specified with the F4 tally (e0 card). The flux results are normalized to 1000 MWth.

**Table 1:** Reactor core parameters used in the F-Lattice analysis^2,3^.

Parameter	CM-BWR
Power (MWt)	1000
Power (MWe)	350
Number of fuel bundles	408
Active fuel height (cm)	274.3
Active poison length (cm)	259.1
Control rod width (cm)	54.8
Control rod thickness (cm)	0.83
Control rod material	B_4_C
Moderator/coolant	H_2_O
Fuel-element array	8 × 8
Assembly pitch (cm)	30.98
Assembly dimension (cm)	13.915 × 13.915
Canister material	SS-304
Canister thickness (mm)	1.535
Clad material	Zircaloy-4
Clad thickness (cm)	0.09072
Fuel-pellet diameter (cm)	1.1088
Pellet-clad gap (cm)	0.0084
Fuel-element pitch (cm)	1.701
Fuel	UO_2_
Fuel enrichment (w %)	2.5

## Results and discussions

### Studying the reactivity of different models of the F-lattice

Firstly, the effective multiplication factor results for the F-Lattice with B_4_C and C are obtained by MCNPX and then they are compared with SCALE-5.1 K-eff results as shown in the previous Table 2. It is expected that the K-eff in case of inserting B_4_C blade (0.93521) is lower than that is produced at withdrawing the B_4_C blade (1.22206). This is due to the high absorption cross section of B-10 which in turn decreases the thermal absorption in the fuel, consequently decreases the number of fission events. Therefore, its K-eff is lower. In case of using carbon, the carbon is considered a good moderator that converts the fast neutrons into thermal neutrons which participate in increasing the fission processes in the fuel. The percent error is calculated relative to SCALE-5.1result. Relative percent differences are calculated through the equation,

$${\text{Relative percent difference }} = { }\left( {\frac{{K_{eff MCNPX - } K_{eff Scale 5.1} }}{{K_{eff Scale 5.1} }}} \right) \times 100.$$

**Table 2:** K-eff for different models of F-lattice calculated by MCNPX and KENO codes.

Model	MCNPX code		Scale-5.1 code		% Error relative to scale 5.1
	K-eff	Sigma	K-eff	Sigma	
4-Bundle (B_4_C)	0.93521	0.00011	0.94341	0.00032	− 0.87
4-Bundle graphite	1.22206	0.0001	1.2077	0.00026	1.19
4-Bundle 10% thinner	0.93883	0.00012	0.946	0.00028	− 0.76
4-Bundle 10% thicker	0.93152	0.00012	0.9392	0.00028	− 0.82

	Rod worth		Rod worth		
4-Bundle (B_4_C)	0.2347		0.2188		7.26
10% thinner	0.2317		0.2166		6.95
10% thicker	0.2377		0.2223		6.48

	Reactivity	Reactivity change			
4-Bundle (B_4_C)	− 0.069278558				
10% thinner	− 0.065155566	0.004122992			
10% thicker	− 0.073514256	− 0.004235699			

It is necessary to interfere that the 4-bundle MCNPX case used 100,000 particles for 250 inactive cycles and 550 active cycles. More particles and histories were used in this case for evaluation of the flux distribution, which requires more information than the k-effective calculations. The SCALE-5.1 case was run with 10,000 particles for 200 inactive cycles and 400 active cycles. The relative percent difference between MCNPX and Scale-5.1 is included to show the level of agreement between the results of the two codes (MCNPX and KENO). This relative difference is shown to be around one percent (0.87% for the B_4_C case and 1.19% for the graphite case).

Secondly, it is also noted that the rod worth of the B_4_C control blades was determined by MCNPX code to be 0.2347. On the other hand, the SCALE-5.1 result leads to a calculated rod worth of 0.2188. Overall, a rod worth of about 0.2347 indicates that the B_4_C control blades (fully inserted) have a very strong effect on the core reactivity. This is beneficial to overall core life, indicating that a gradual removal of the control blades from the core will allow for a prolonged fuel burn. The calculated relative percent error for this case is 7.26%.

Thirdly, the variation in the thickness of the B_4_C control blade has an impact of the bundle reactivity. Studying this parameter (variation of thickness) confirms that MCNPX code is capable of calculating the effective multiplication factor with less than one percent error for the F-Lattice when the thickness of the control blade is increased or decreased by 10%. In these two models, the bundle pitch remains constant but the thickness of the control blade is varied by ± 10%, thus affecting the size of the water gap between the fuel and the control blade. The variation of the thickness of the control blades also results in a significant change in the amount of control material present in the bundle.

According to the results, it is observed the 10% thinner model presents higher K-eff (0.93883) than that of 10% thinner (0.93152). The 10% thinner case leads to an increase in the effective multiplication factor by 0.362% from nominal case (B_4_C case). The 10% thicker case produces a decrease by nearly the same factor (0. 369%). The (±) 10% thickness variation leads to overall reactivity changes of about (±) 0.004. Relative error for MCNPX compared to SCALE-5.1 remained below one percent for this variation (0.76 and 0.82) for − 10% thinner and + 10% thicker respectively.

Fourthly, the rod worth calculations were carried out because of the significant increase or decrease in control material volume. From the tabulated data, it is obvious that rod worth is directly proportional to the ratio of control blade surface area to fuel volume. And the fuel volume is constant. Therefore, the rod worth is directly proportional to the control blade area. The rod worth is higher for the 10% thicker. The relative percent error is around 7% (6.95% for thinner case and 6.48% for thicker case).

Finally, Reactivity changes are expected to be small due to the fact that the control blades’ absorption cross-section, the control blade surface area and the fuel volume are not significantly affected in this parametric study. The three cases of nominal control blade thickness of 8.3 mm, 10% thicker (9.13 mm) and 10% thinner (7.47 mm) are studied with MCNPX code with 50,000 particles, 250 inactive cycles and 350 active cycles.

### Comparison between the thermal, epithermal, fast and total flux distribution of the F-bundle in case inserting and withdrawing the B_4_C control blade

In this section, the flux distribution is studied over the fuel bundle in case of using B_4_C (nominal case) and C in the control blade. This is carried out by using Fmesh card (used in MCNP6) that allows dividing the rectangular assembly into a number of meshes. As the number of meshes in the three dimensions of the geometry increases, more definite flux shapes are obtained. In the models under investigation, 10 meshes are used in the X, Y and Z axes. The peak and average flux are calculated and compared with SCALE-5.1 results. The exhibited behavior of flux distribution is XY-plane surface contour and the Z-plane represents the flux values. The neutron energy grouping was 0–0.225 eV for thermal neutrons, 0.225 eV–0.1 meV for epithermal neutrons and 0.1 meV and above for fast neutron. All the flux values are normalized to 1000 MWt (the power of the reactor core). The real flux in (n/cm^2^ s) can be evaluated from the following equation^14^.

$${\text{Neutron flux }}\left( {{\text{n}}/{\text{cm}}^{2} \,{\text{s}}} \right){ } = {\text{ F}}4{\text{ tally }}\left( {1/{\text{cm}}^{2} } \right) \times { }\frac{P \times \nu }{{ Q \times K_{eff} }},$$

where P = Thermal reactor power in MW (1000 MWt), Q = Energy released per fission, MeV (200 meV), υ = Average number of neutrons released per fission, determined by MCNPX code, K-eff = Effective neutron multiplication number, determined by MCNPX.

The neutron flux profile is an important characteristic in determining the behavior of the reactor core and understanding the isotopic composition and the contributions from Plutonium. For these two purposes, the thermal, epithermal and flux profiles for the BWR bundle are plotted and analyzed^15^**.**

**Figure 2:**
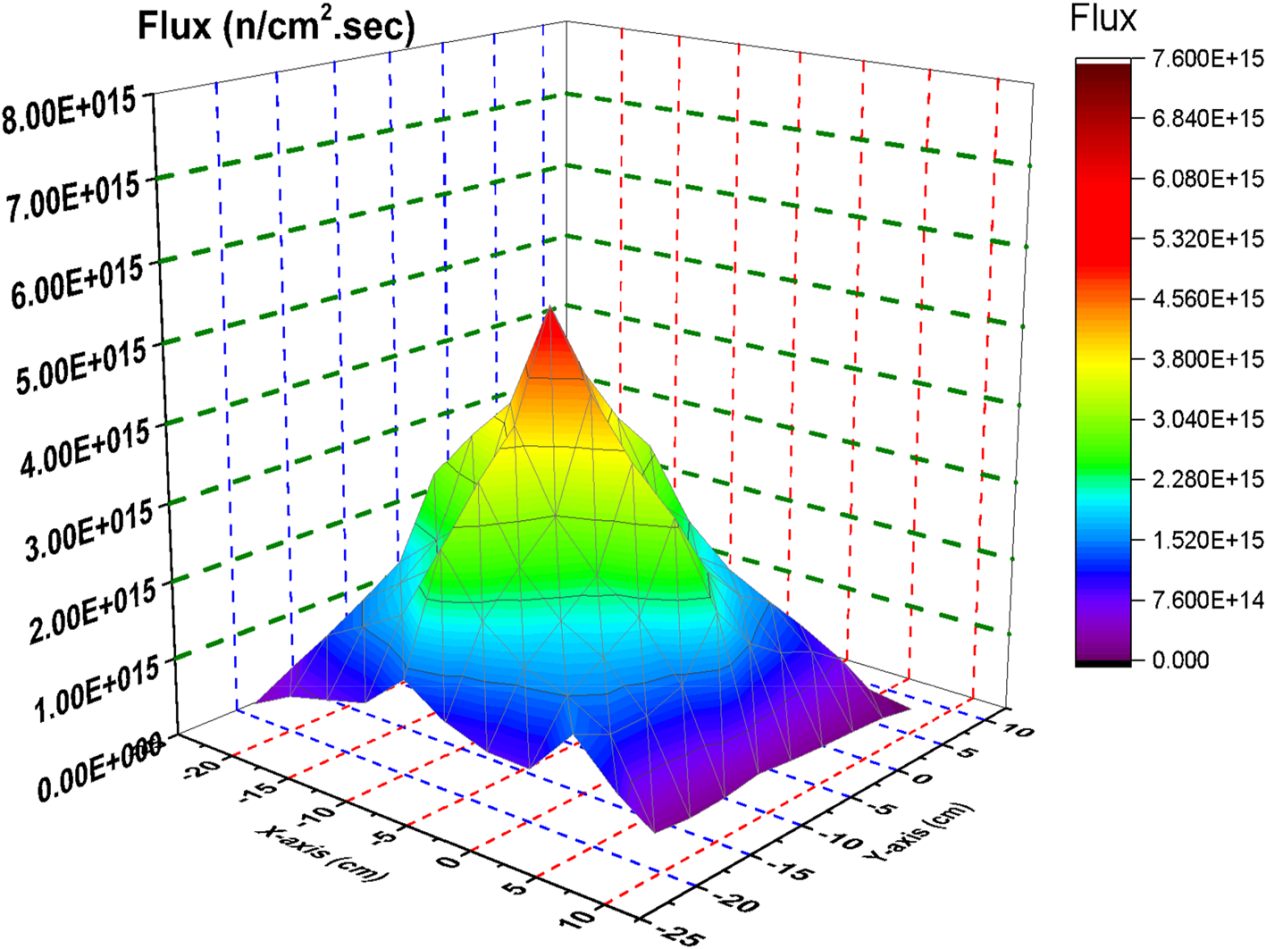
Thermal Flux distribution for the F- bundle using 10 meshes in case of B4C in the control blade.

**Figure 3:**
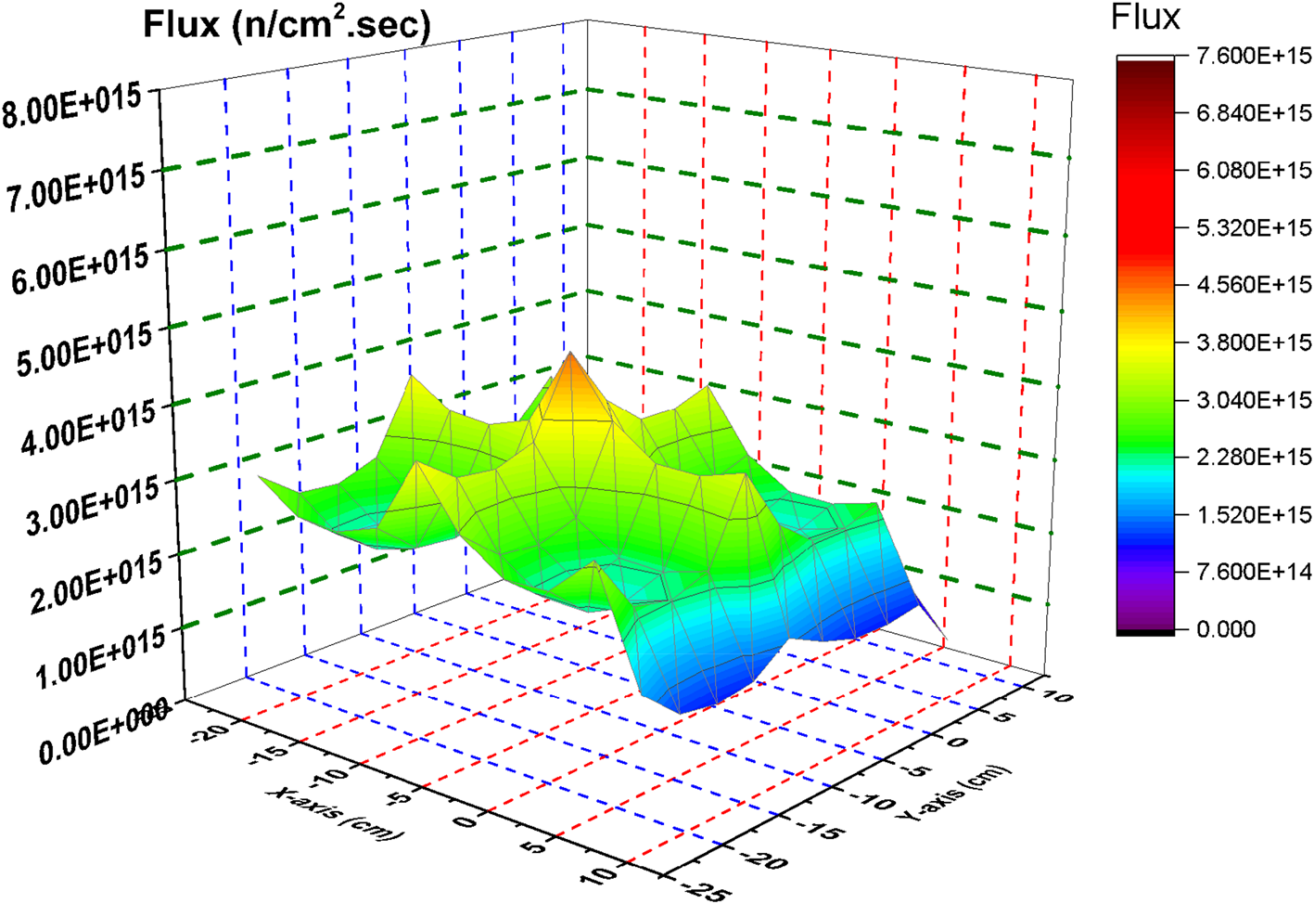
Thermal Flux distribution for the F-bundle using 10 meshes in case of C in the control blade (withdrawing B_4_C).

From Fig. 2, there is a decrease in the thermal flux values at the bundle periphery due to the presence of B_4_C that absorbs thermal neutrons. The peak of the thermal flux is found in the middle of the bundle owing to the presence of water that moderate fast neutrons to thermal neutrons. On the other hand, in Fig. 3, the thermal flux peaks found in that figure are located near the graphite control blade that does not absorb thermal neutrons. A central thermal flux peak is also found in the middle of the bundle due to the reason mentioned before. The very sharp decrease in both figures towards the Y-axis in blue and violet colors is due to locating the mesh cells of this part outside the bundle. This part summarizes that replacing B_4_C with graphite would actually result in a larger power level, and also larger flux values. 1000 MWt was maintained in both cases to allow for a direct comparison of the effect the material change has on the flux distribution. The constant power normalization is the reason that the peak flux values in the thermal flux (Fig. 2) are larger for the nominal case, compared to the graphite case (Fig. 3).

**Figure 4:**
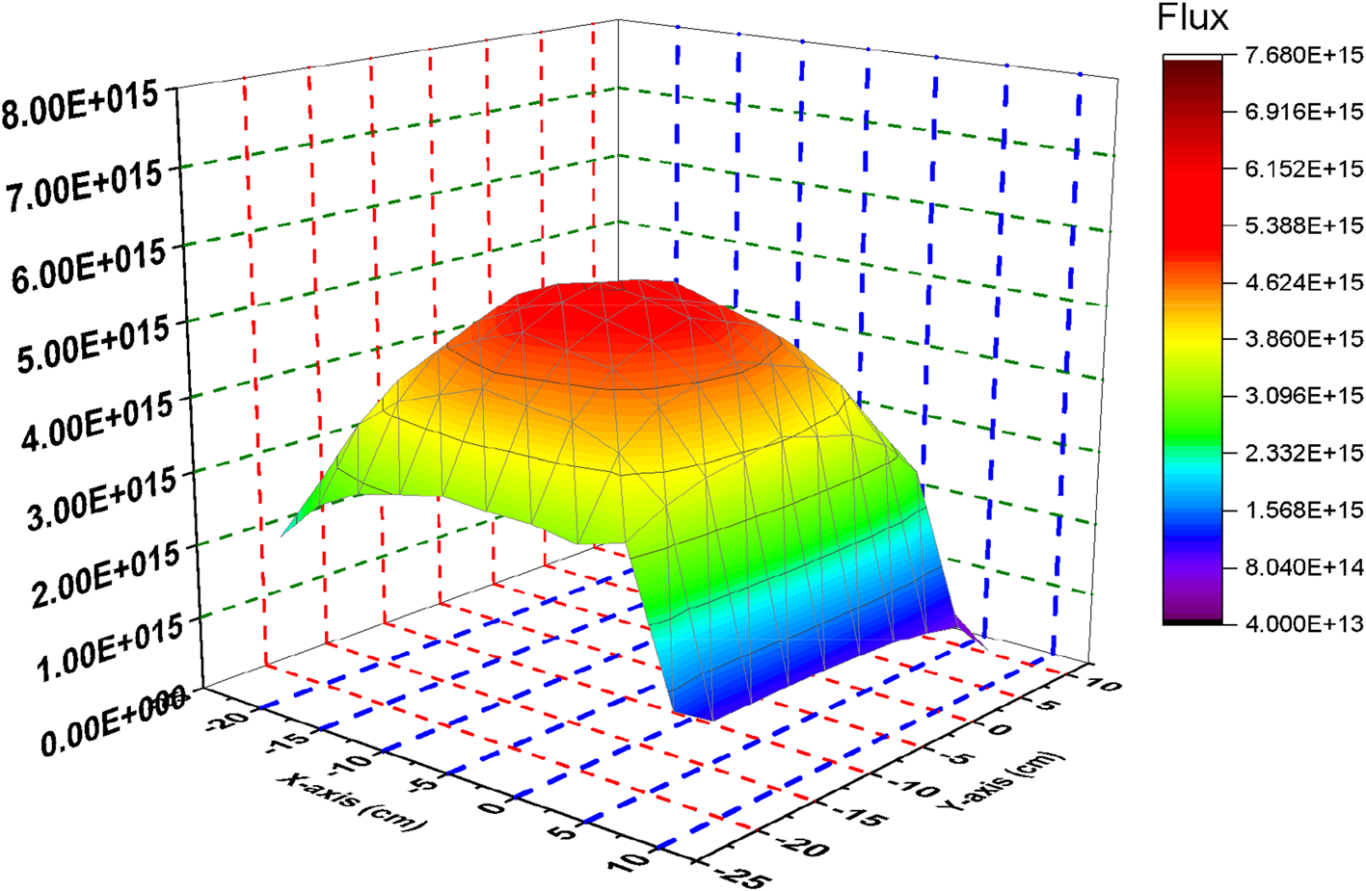
Epithermal Flux distribution for the four bundle using 10 meshes in case of B_4_C in the control blade**.**

**Figure 5:**
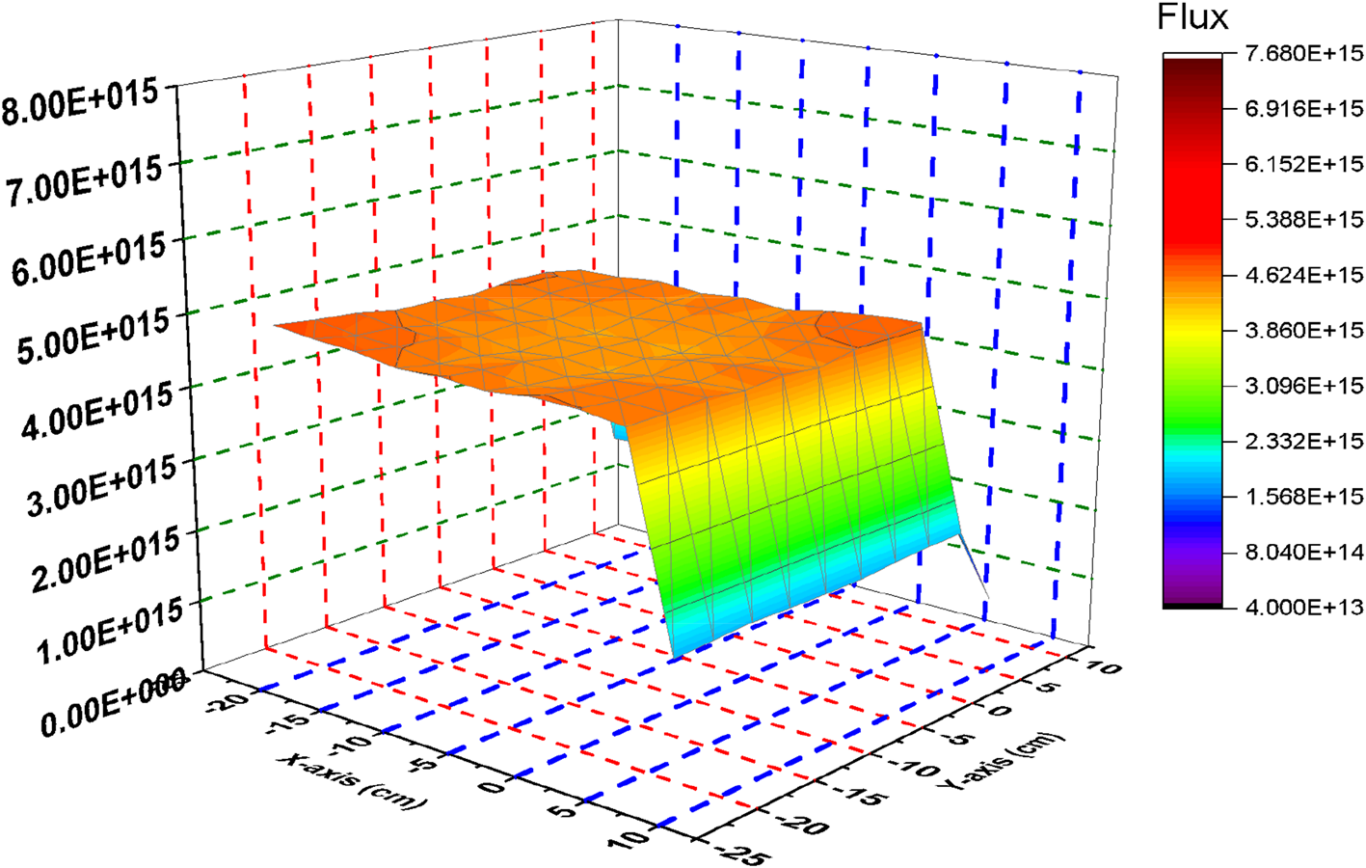
Epithermal Flux distribution for the four bundle using 10 meshes in case of C in the control blade**.**

Figures 4 and 5 show that there is a significant difference in the epithermal flux distribution in the two cases under study. It is clear that the graphite case contributes to flat and lower epithermal flux profile through the bundle than the B_4_C case. This may be attributed to the interaction of fast neutrons with carbon and this resulting in lower epithermal flux over the F-lattice. For the B4C model, the absorption of thermal neurons causes plenty of epithermal and fast neurons over the bundle fuel.

**Figure 6:**
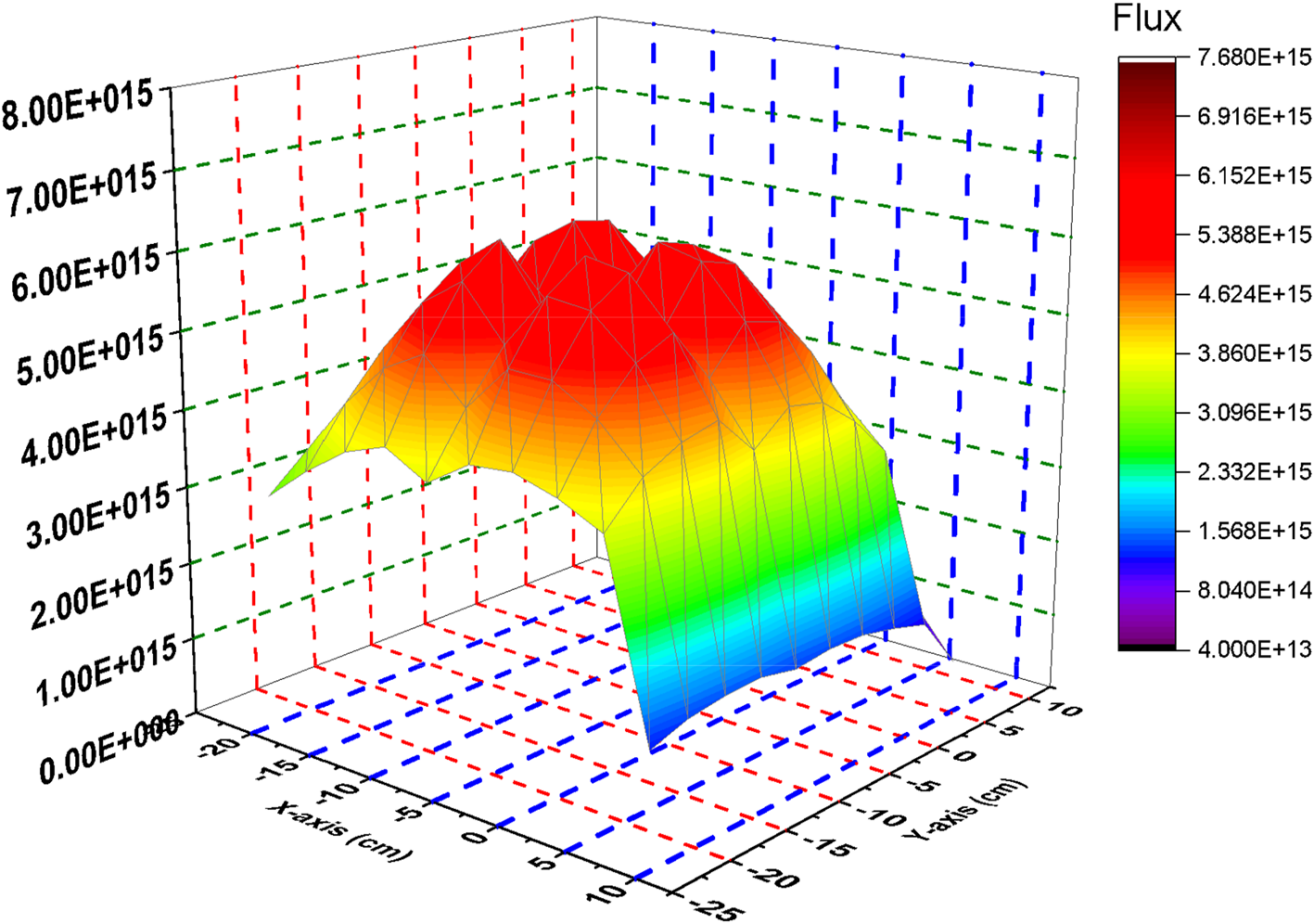
Fast Flux distribution for the four bundle using 10 meshes in case of B4C in the control blade.

Figure 6 gives the fast flux distribution in case of B_4_C case. Four fast flux peaks are found in the figure. Each peak corresponds to a fuel bundle. This means that decreasing the fission reactions in the four bundles contributes in increasing the number of fast neutrons over the four bundles. A very sharp decrease in the fast flux profile is also found because of the void mesh cells located outside the F-lattice.

**Figure 7:**
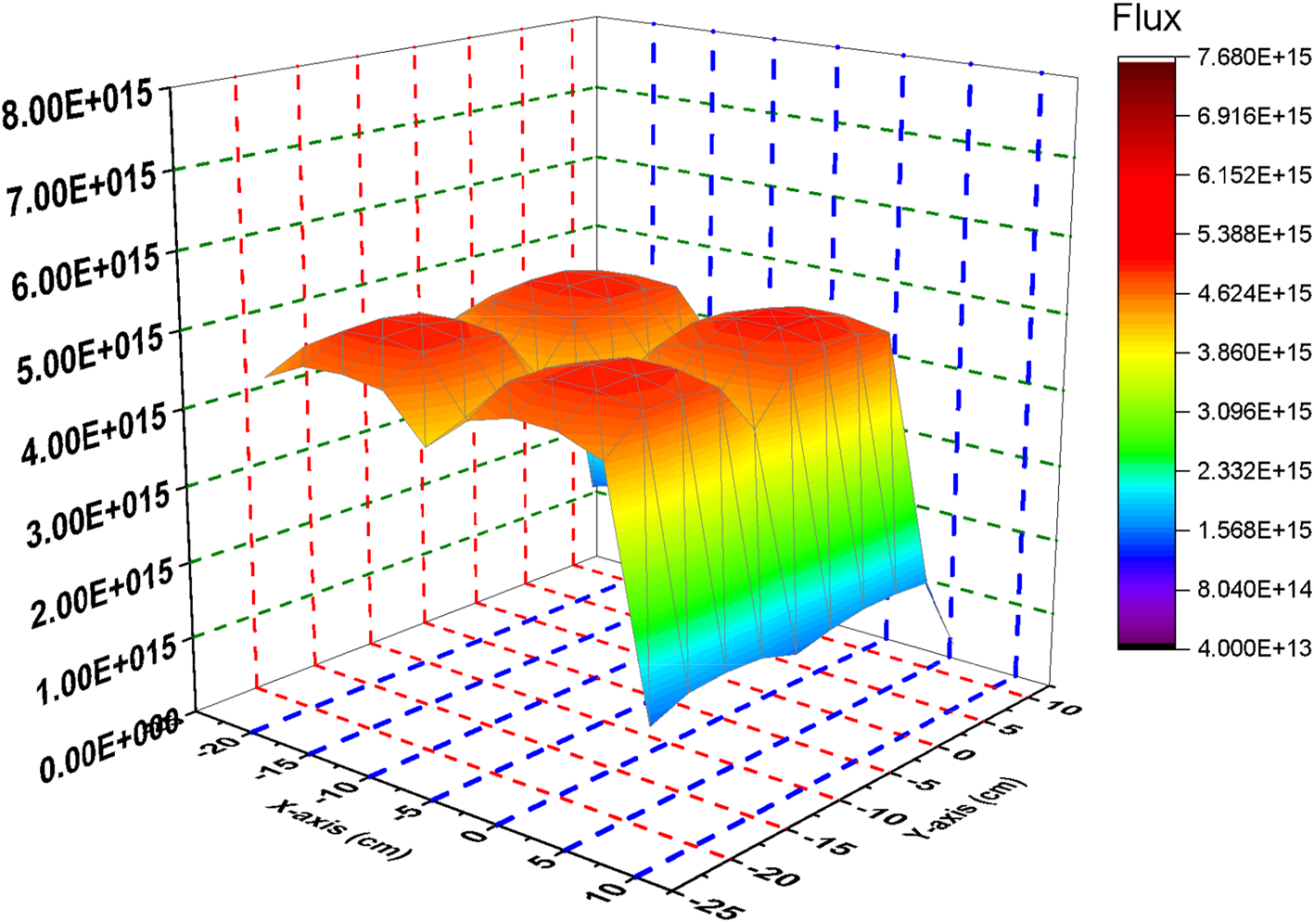
Fast Flux distribution for the four bundle using 10 meshes in case of C in the control blade.

The behavior of fast flux profile at withdrawing the B_4_C is nearly similar to that is obtained in case of inserting B_4_C but the values are lower in the carbon case. The scattering of fast neutrons by carbon around the bundle makes fast neutrons to be lower in the F-lattice (Fig. 7).

**Figure 8:**
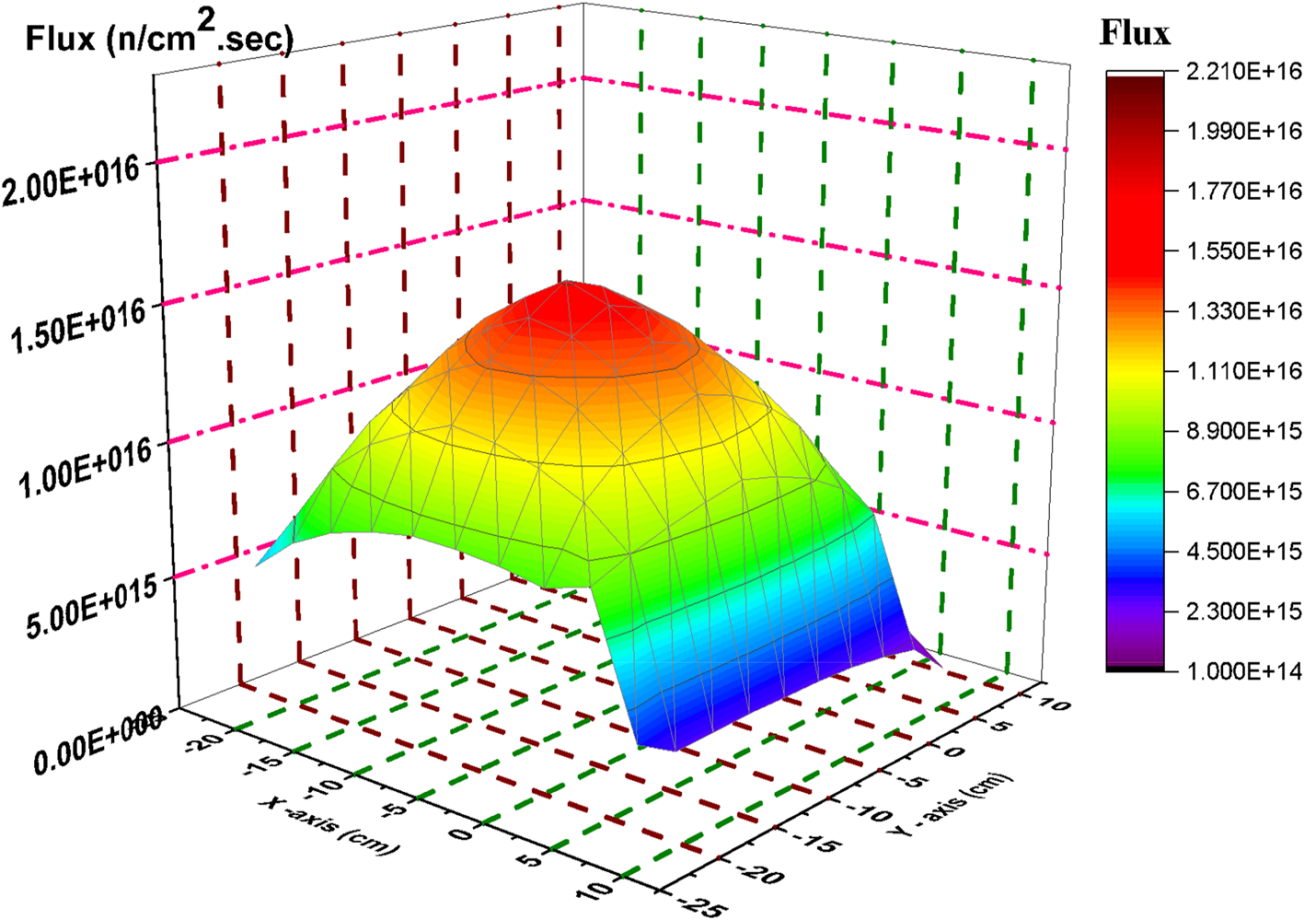
Total flux distribution for the four bundle using 10 meshes in case of B_4_C in the control blade.

**Figure 9:**
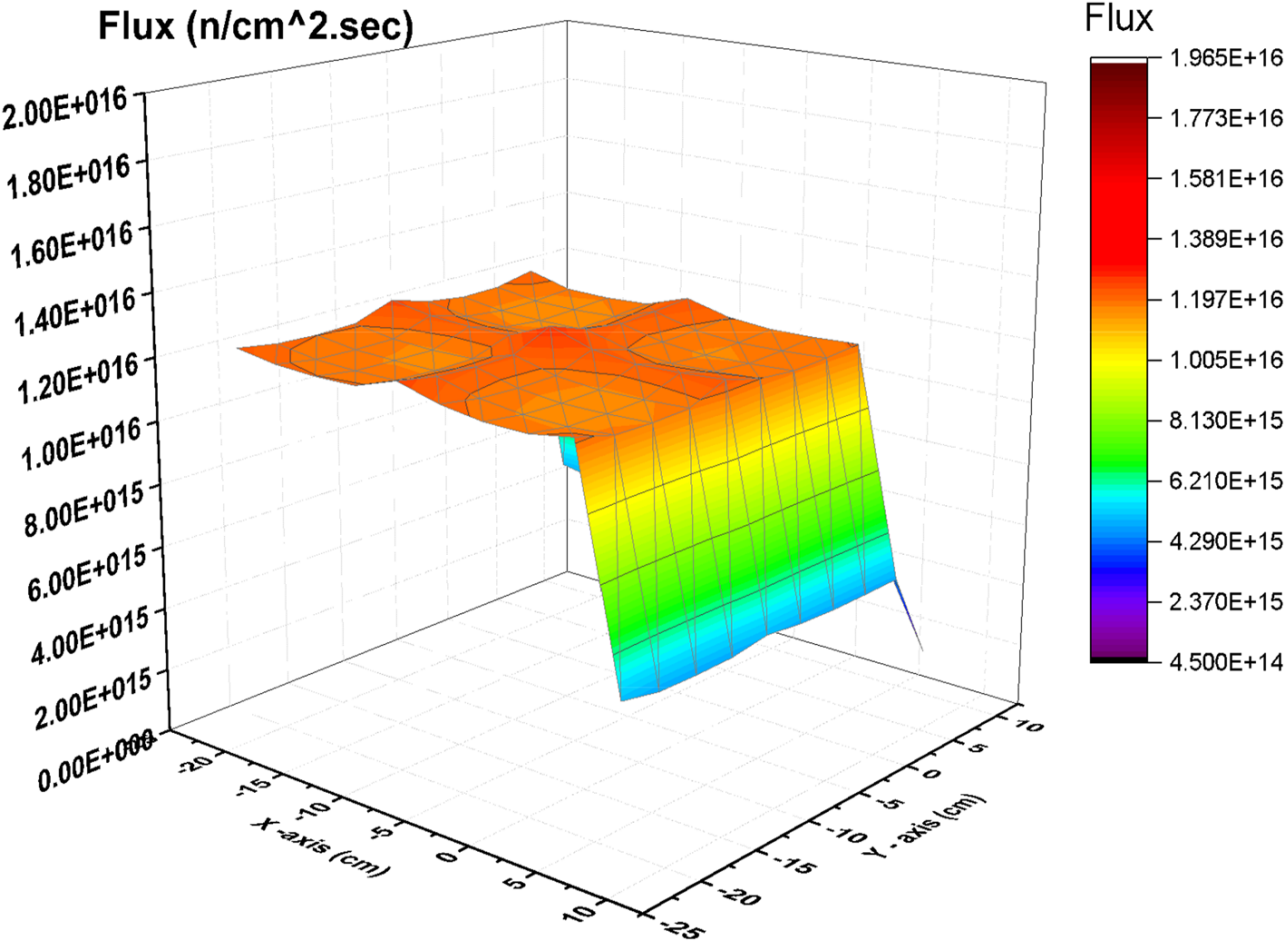
Total flux distribution for the four bundle using 10 meshes in case of C in the control blade**.**

Figures 8 and 9 give the total flux profile which is the combination between thermal, epithermal and fast flux. The total flux is essentially flat for Carbon model.

The peak flux estimated by MCNPX in case of B_4_C model is 2.20188 × 10^16^ and it is found to be 2.1224 × 10^16^ estimated by Scale-5.1 (Ref^1^). This results in a relative percent error 3.74%.

### Comparison between the thermal, epithermal, fast and total flux distribution over the B_4_C and C in the blades

Flux distribution is also calculated through the control blade of a rectangular shape located in the upper of the F-bundle. The dimensions of this blade are 0.415 cm × 27.4 cm × 259.2 cm. Also, all flux values are scaled to units of the number of neutrons per centimeter squared per second to a power level of 1000 MWt. 10 meshes are taken for the calculations.

**Figure 10:**
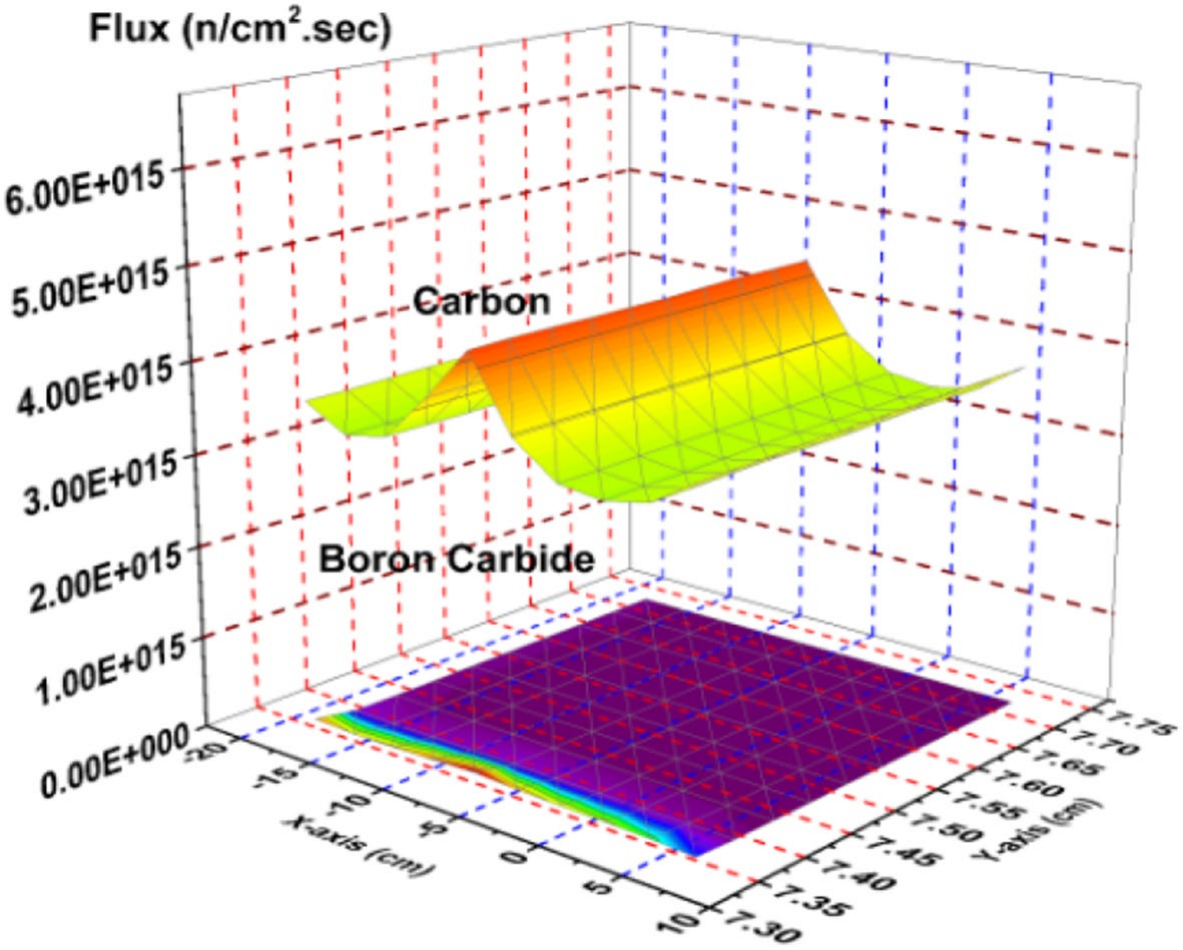
Thermal flux distribution in the carbon and boron carbide control blades.

Thermal neutron flux shown in Fig. 10 provides the most relevant example of the model’s rod worth. The strong absorber, B_4_C results in near zero thermal neutron flux through the control blade. The stronger moderator, graphite, is shown to maintain a high thermal neutron flux throughout the blade, which is directly related to the significantly larger k-effective associated with the graphite system.

**Figure 11:**
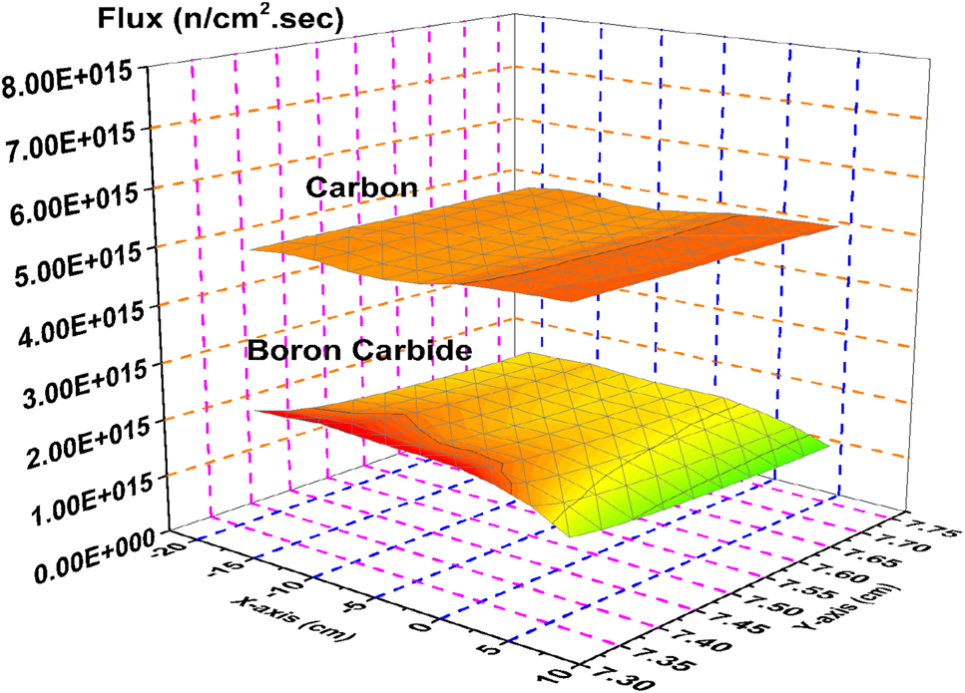
Epithermal flux distribution in the carbon and boron carbide control blades.

Figure 11 is a plot of the epithermal neutron flux. The graphite system presents a larger flux profile through the blade than the B_4_C system. This is expected as more of the fast neutrons are slowed down via interactions with the graphite than are by B_4_C.

**Figure 12:**
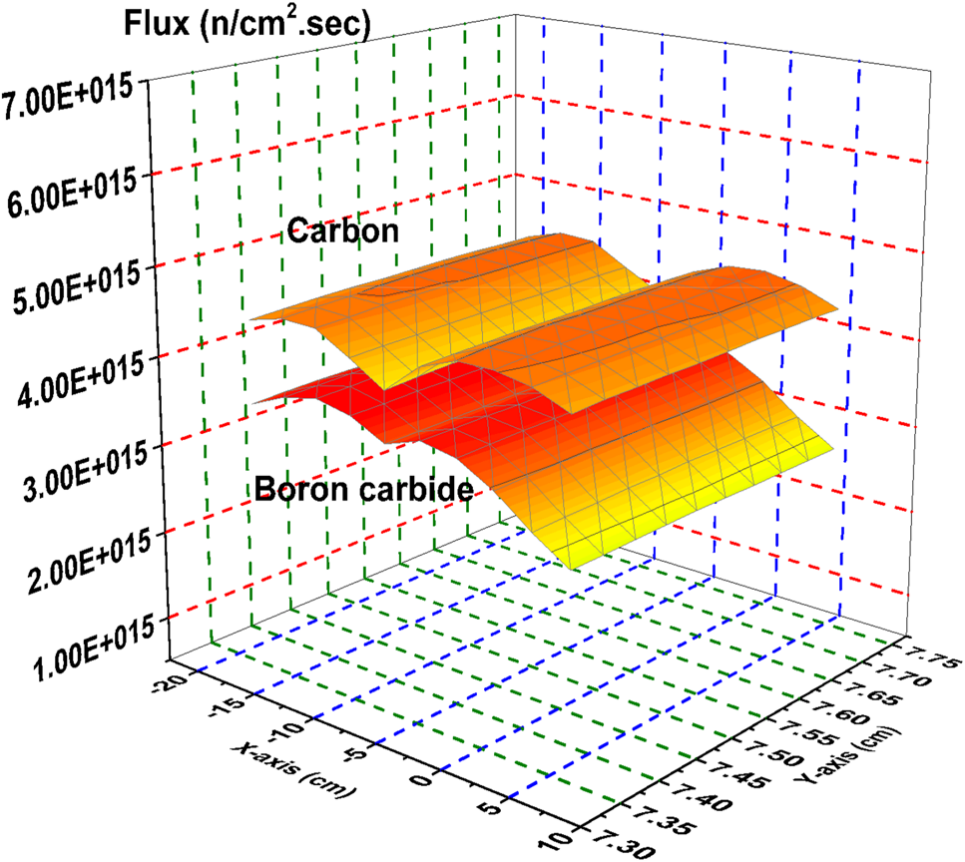
Fast flux distribution in the carbon and boron carbide control blades.

Figure 12 provides the flux profile for the fast neutrons. As expected, the graphite system again presents larger flux values, though it is not as significant as it is in the epithermal or thermal cases. Slightly more fast neutrons are present in the graphite case due to the increase in overall fission reactions that are taking place compared to the B_4_C control rod system.

**Figure 13:**
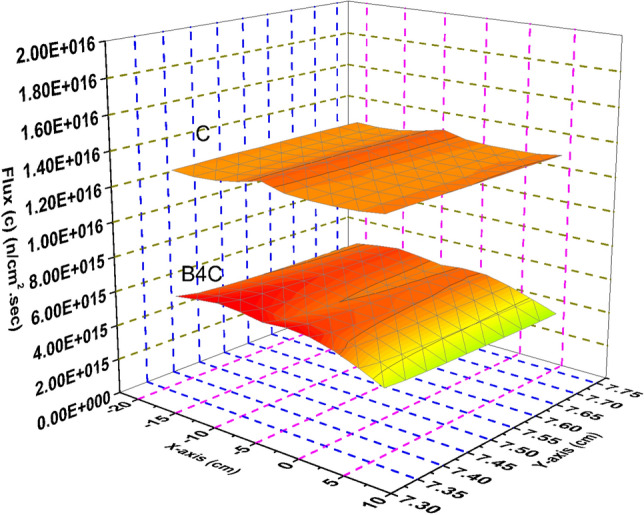
Total flux distribution in the carbon and boron carbide control blades.

There is significant difference in the total neutron flux (Fig. 13) between the graphite case and the nominal B_4_C case, mostly due to the increase in the thermal and epithermal neutron population shown in Figs. 10 and 11.

### Burnup calculations for the 4-bundle model

Burn-up analysis is important to determine the core life, as well as how the power and flux distributions change with time. During the reactor operation, fuel composition changes in terms of quantity of fissile, fertile and poisoning isotopes. Fissile isotopes as U-235 and Pu-239 are depleted continuously as fissions take place, while fertile isotopes as U-238 and Th-232 are transformed into Pu-239 and U-233 respectively^16^. The production of poisonous isotopes, such as Xe-135, quickly accumulates and reaches to an equilibrium state, which in turn helps in regulating the reactivity of the core. At the same time, fission products, such as Cs-137, are produced. All of these reactions have an effect on core life, power distribution, flux distribution and reactivity. How the core changes with time is an important aspect of any reactor and must be investigated in detail. 75 fission products and 24 actinides were tracked in the MCNPX depletion calculations^17^.

Two tests were carried out on the F-Lattice, assuming a fresh core with an average burn rate of 9.80 MWt (control blades remain fully inserted over the entire duration of burn). The first burn-up calculation was performed for 1 month (31 days). MCNPX code performed 31 burn steps (1 day intervals), which refers to the time step in which neutron fluxes are actualized with time.

The second test was performed for 20 months with 40 steps (2 week time steps) and 40 internal steps. K-effective calculations are only presented in this test. (For a preliminary study, and due to time constraints, MCNPX simulations were run with reduced accuracy, using only 10,000 particles for 150 inactive cycles and 150 active cycles. The obtained results by MCNPX code are compared with those obtained by (MCNP5—Monteburns) combination. The idea of coupling MCNP5 with Monteburns in reference^1^ depends on calculating the concentrations of fuel isotopes at the required irradiation value of the full core. Then the new atom densities are transferred to MCNP5 file to calculate the desired parameters as k-eff and power distribution at the stage under study^18^. The uranium enrichment, the boron enrichment in the control blade and the volume of moderator in the reactor core are important factors which play an important role in determining the k-eff value and the flux distribution shape^19^.

### The results of the 1 month burn-up calculation with 1 day time step

**Figure 14:**
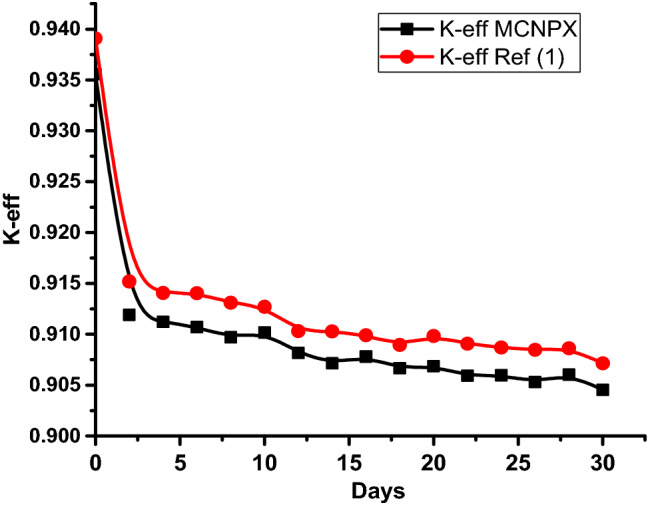
K-effective results for 30 day burn with 1 day time steps.

**Figure 15:**
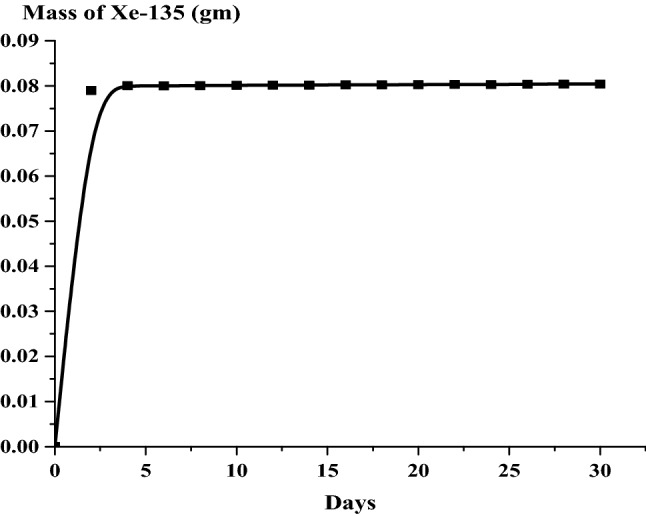
Grams of Xe-135 in the bundle over 30 days with 1 day time steps.

**Figure 16:**
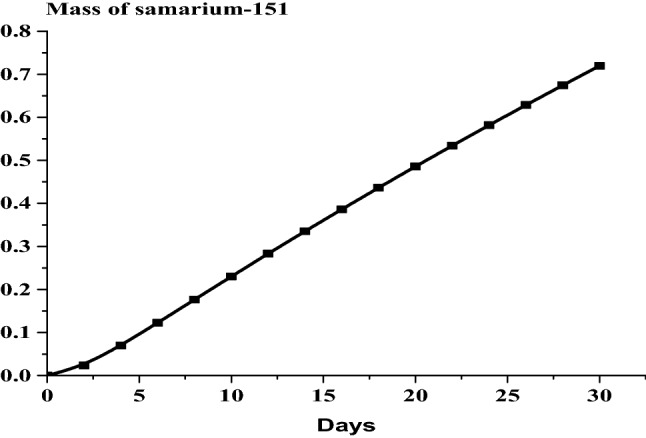
Grams of Sm-151 in the F-lattice over 30 days with 1 day time steps.

**Figure 17:**
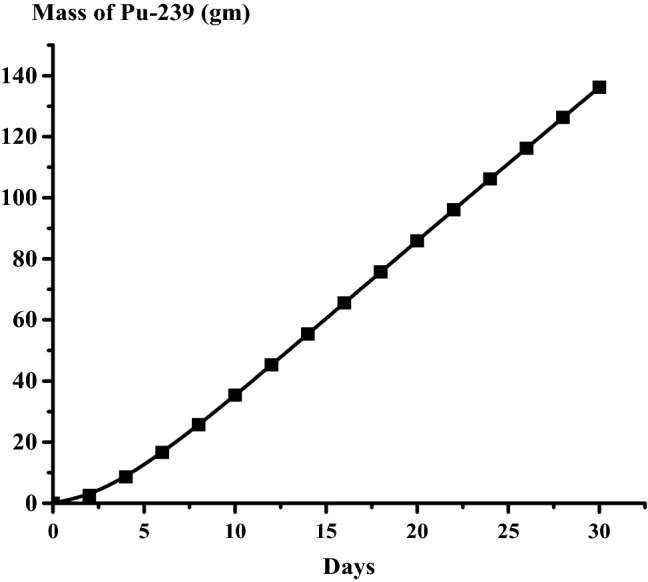
Grams of Pu-239 in the bundle over 30 days with 1 day time steps.

**Figure 18:**
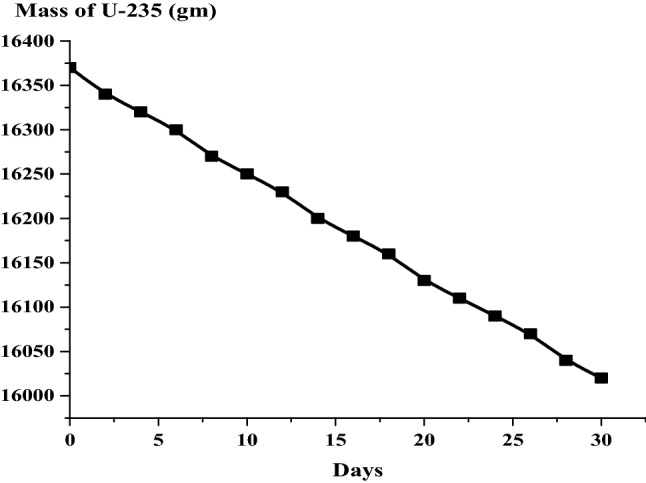
Grams of U-235 in the F-lattice over 30 days with 1 day time steps.

**Figure 19:**
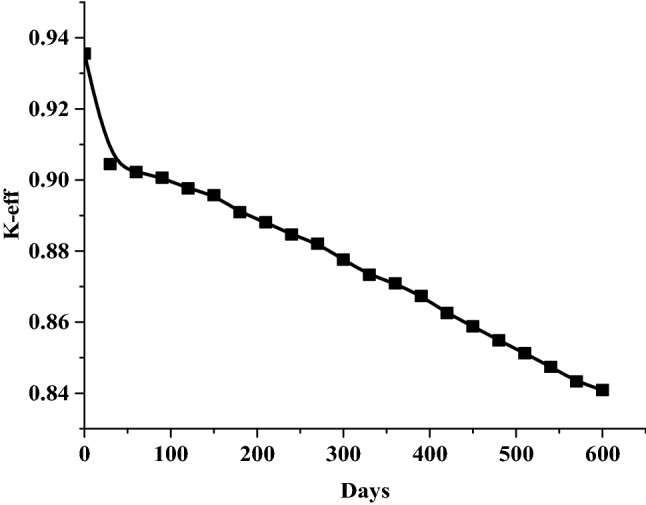
K-effective results for 20 months burn with 2 week time steps.

The large drop in k-effective on the first day of burn is a result of the production of Xe-135 present in the reactor (Fig. 14). This well known Xenon poisoning which in turn has a fast and dramatic effect on the core reactivity before it reaches an equilibrium state at which the mass of Xenon is nearly 0.08 gm as in Fig. 15. Fission product accumulation of Sm-151 and Pu-239 are linear as in Figs. 16 and 17. The mass of U-235 decreases linearly (Fig. 18). Results of K-eff for 20 month burn with 2 week time steps are depicted in Fig. 19.

The final k-effective of Reference results, at the end of 30 days, is 0.9072, a 3.3% decrease from the initial value of 0.9380. But for MCNPX results, the final value (0.9455) decreases by 3.1% from the initial value (0.93556). For the K-eff results for the 20 month burn, with 2 weeks time steps, the final k-effective value is 0.8441, a 10.0% decrease from the initial value of 0.9380 in the reference result. On the other hand, for MCNPX results, the final value (0.8409) decreases by 9.47% of the initial value (0.93556).

### Burnup calculations of F-lattice to 70 GWd/ton (4.679E+03 days)

After the previous validation of MCNPX code with (MCNP5-Monteburn) results at 30 and 600 days is verified, the fuel F-bundle is burnt to 70 GWd/ton. The burnp calculations are performed at inserting the B_4_C blade, withdrawing it, using + 10% thinner B_4_C and − 10% thicker B_4_C. A comparison is held between the four cases from the view of effective multiplication factor and mass of U-235. Additionally, the flux profile is plotted for the B_4_C and C cases to determine the favorable distribution of thermal flux over the bundle that serves in stabilizing the reactor operation at the end of life.

**Figure 20:**
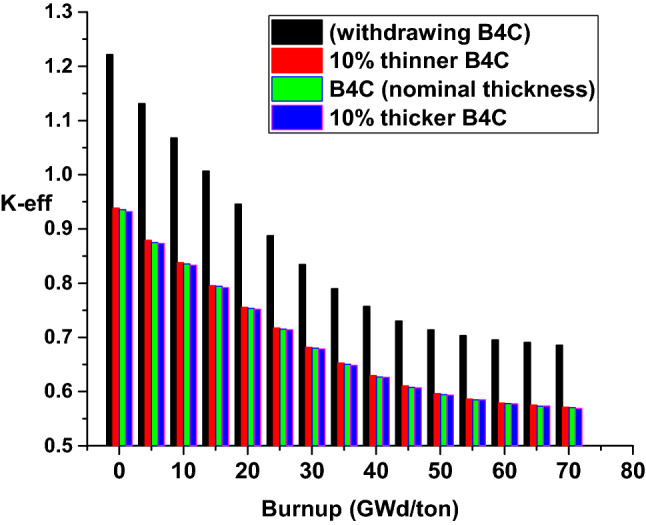
K-effective results with burnup for the four cases.

**Figure 21:**
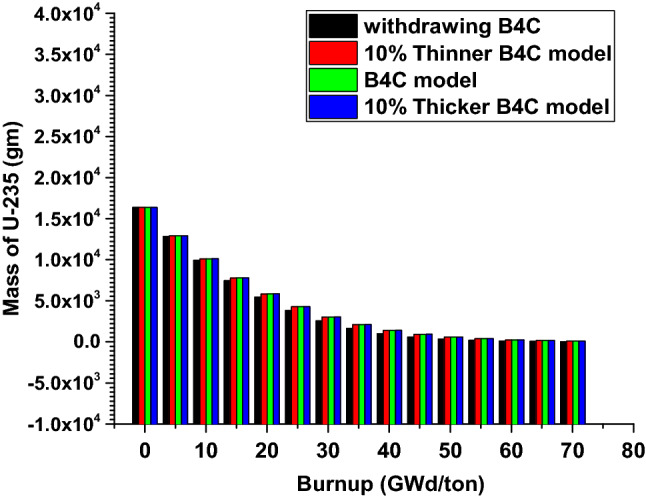
Mass of U-235 with burnup for the four cases.

As observed in Fig. 20 and Table 3, the highest values of K-eff are obtained at withdrawing the B_4_C blade. The lowest values are for 10% thicker of B_4_C. This shows that as the content of absorbing material increases, the reactivity decreases. Figure 21 and Table 4 depict the variation of U-235 mass with time. In all cases, the mass decreases with time. From the tabulated results, the mass of U-235 at 70 GWd/ton for B_4_C and 10% thicker cases is higher than that of the other cases (C and 10% thinner). Therefore, those two models achieves the longest life cycle among the 4 models.

**Table 3:** Numerical values of K-eff with burnup for the 4 cases.

Burnup	Withdrawing B_4_C	10% Thinner B_4_C model	B_4_C model	10% Thicker B_4_C model
0.00E+00	1.22193	0.93838	0.93556	0.93192
5.00E+00	1.13131	0.87913	0.87479	0.87339
1.00E+01	1.06823	0.83776	0.83556	0.83297
1.50E+01	1.00688	0.79561	0.79423	0.79166
2.00E+01	0.94602	0.75546	0.75321	0.75168
2.50E+01	0.88782	0.7176	0.71527	0.714
3.00E+01	0.83467	0.68167	0.67997	0.67846
3.50E+01	0.78985	0.65287	0.65032	0.64836
4.00E+01	0.75719	0.6295	0.62666	0.62622
4.50E+01	0.73052	0.61046	0.60803	0.60689
5.00E+01	0.71419	0.59639	0.59441	0.59322
5.50E+01	0.70358	0.58637	0.58479	0.58489
6.01E+01	0.69538	0.57902	0.57785	0.57743
6.51E+01	0.69081	0.57517	0.57304	0.57339
7.01E+01	0.68586	0.57131	0.57049	0.56879

**Table 4:** Mass of U-235 at the different stages of burnup for the 4 cases.

Burnup (GWd/ton)	Withdrawing B_4_C	10% Thinner B_4_C model	B_4_C model	10% Thicker B_4_C model
0.00E+00	16,370	16,370	16,370	16,370
5.00E+00	12,840	12,920	12,920	12,920
1.00E+01	9931	10,110	10,110	10,120
1.50E+01	74,480	7771	7778	7786
2.00E+01	5460	5838	5847	5857
2.50E+01	3825	4269	4276	4287
3.00E+01	2562	3032	3037	3049
3.50E+01	1641	2091	2096	2105
4.00E+01	1009	1404	1407	1417
4.50E+01	600.7	922.3	925.1	932.5
5.00E+01	350.2	595.3	598.3	604
5.50E+01	201.6	379.9	382.3	3867
6.01E+01	115.1	240.7	242.8	246
6.51E+01	65.86	152	153.7	156.2
7.01E+01	37.83	96.1	97.27	99

### Flux distribution for C and B_4_C models at 70 GWd/ton (4.679E+03 days)

At the end of reactor operation, it is very important to study how the thermal flux is distributed at inserting and withdrawing the B_4_C blade. Thermal flux profiles are plotted by the way illustrated before.

**Figure 22:**
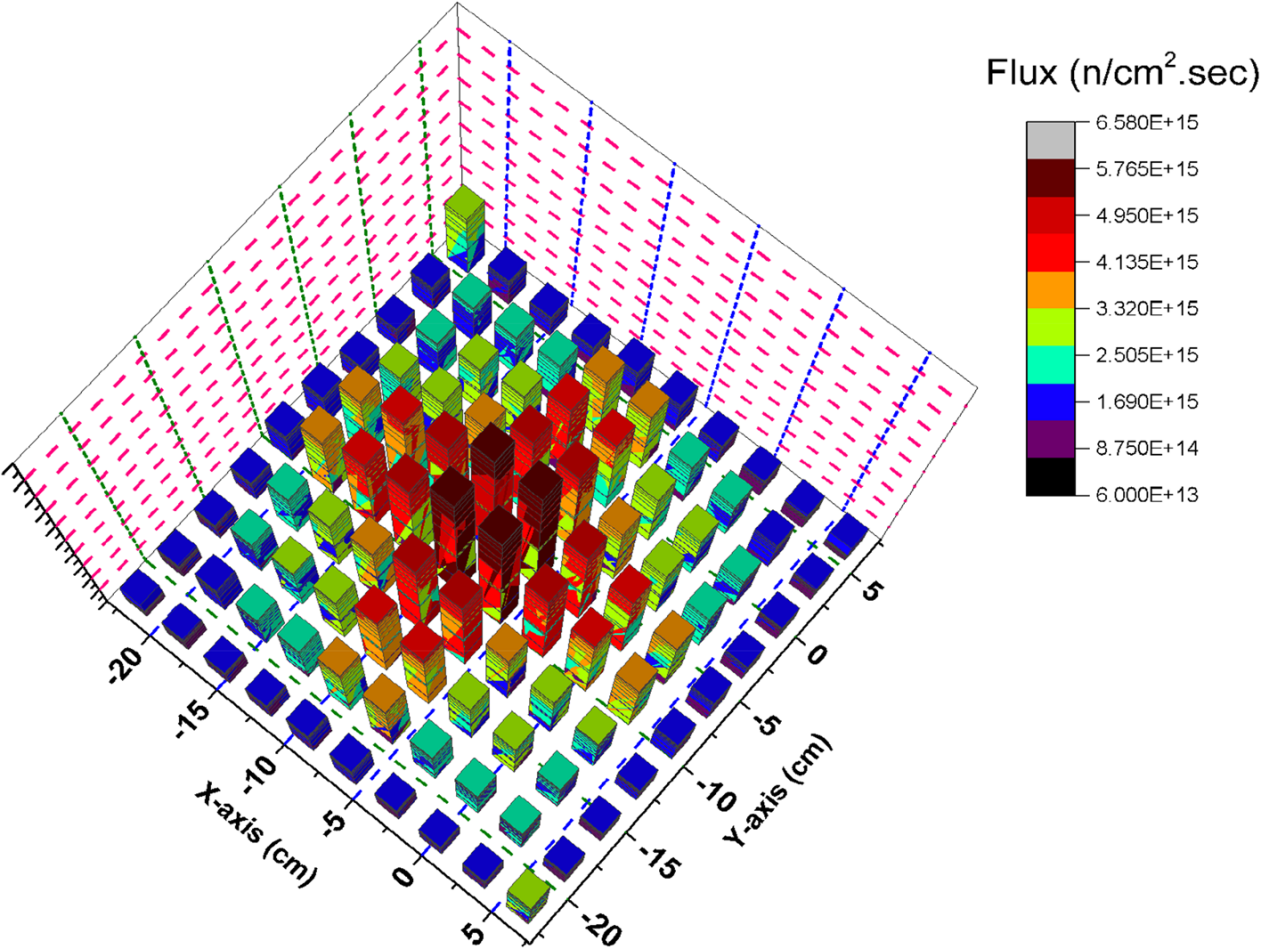
Thermal flux distribution at 70 GWd/ton for the B_4_C model.

Figure 22 show the spatial thermal neutron flux for the B_4_C model at the end of cycle. Based on these results, we can see the trend where maximum flux is in the centre of the bundle and gradually reduced towards the edges due to the presence of B_4_C that absorbs thermal neutrons. The significant peak at the bundle centre is also because it has larger water volume compare to other locations in the geometry. Hence more moderation process occur which increased thermal neutron flux at the centre of F-lattice. Therefore, it is obvious from the figure that the thermal neutron flux has no fluctuations in the bundle that is resulted from uniform power distribution over each bundle.

**Figure 23:**
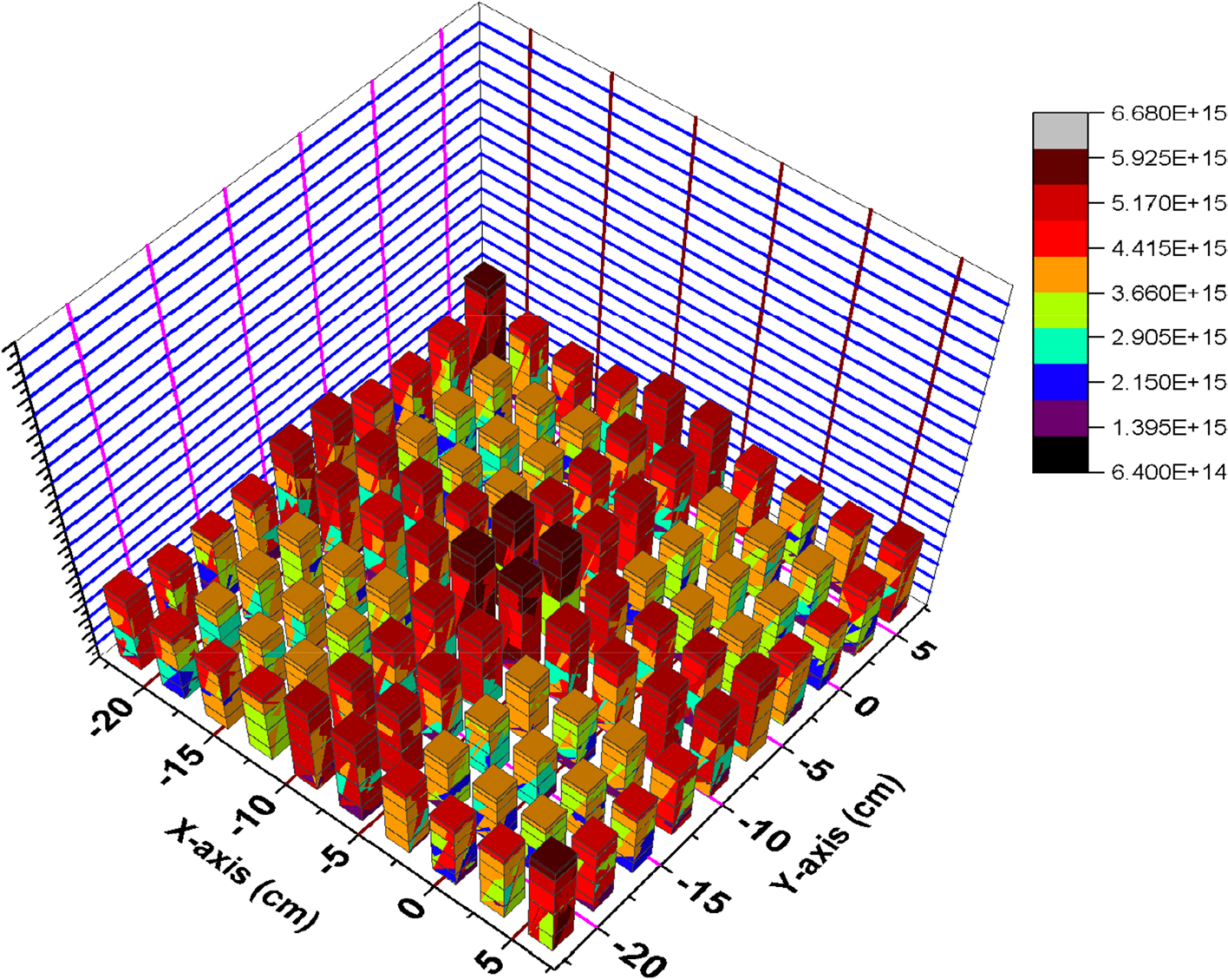
Thermal flux distribution at 70 GWd/ton for the C model.

At withdrawing the B_4_C (Fig. 23), the thermal flux fluctuations are observable due to the presence of carbon that participates in decreasing the thermal flux over the four bundles and increasing it at the edges and the middle. So, the radial thermal flux profile was found to be non-cosine shape. Finally, it can be said that the B_4_C model achieves more safety and long operation for the Boiling Water Reactor. Increasing the ratio between the average flux and maximum flux is evidence that a flattening behavior of thermal neutrons is obtained for the B_4_C model. At the beginning of life in Fig. 2, ɸ_average_/ɸ_maximum_ = 1.66302 × 10^15^/7.59477 × 10^15^ = 0.2189 and the same ratio is 1.94 × 10^15^/6.57 × 10^15^ = 0.2956 at the end of life .

## Conclusion

MCNPX is found be capable of modeling and simulating the BWR F-lattice. Four cases are investigated. These cases include inserting, withdrawing, using 10% thinner and using 10% thicker of B_4_C. A comparison is held between the four cases from the point of view of multiplication factor, reactivity and rod worth. The results showed the role of B_4_C control blade in decreasing the reactivity of the bundle. Spatial thermal, epithermal and fast fluxes are studied for the B_4_C and C cases. This is carried out by MCNP6 found in MCNPX. The flux results summarized that thermal flux over the B_4_C material is lower at the bundle periphery and is peaked at the centre but for the C material, thermal flux peaks are found at the periphery due to the moderation effect of carbon. For investigating which model that provides long life cycle and flatting thermal flux at the end of operation, depletion calculations are conducted till 70 GWd/ton. It was clear that the B_4_C and 10% thicker models provide prolonged life cycles due to the high amounts of U-235. The results also concluded that the flattening of thermal flux is observed for B_4_C model and this was predicted via ɸ_average_/ɸ_maximum_. This gives high stability and safety for the BWR operation.

## Supplementary Information

Supplementary Information.
