# Supplementary material for: The effect of control rods on the reactivity and flux distribution of BWR 4 bundle using MCNPX Code
Source: Sci Rep. 2021 Apr 22;11:8713. doi: 10.1038/s41598-021-88067-0 (PMC8062693; doi:10.1038/s41598-021-88067-0)
Supplement: Supplementary file 1 — Supplementary Information. [file 41598_2021_88067_MOESM1_ESM.docx]

**Supporting Information**

The Fmesh card can be written as following in MCNP6 code

Fmeshn:pl GEOM=geo ORIGIN=0,0,0

imesh=iii iints=III

jmesh=jjj jints=JJJ

kmesh= kkk kints=KKK

n=tally number (can only be tally type 4)

pl= particle type, N, P or E

Geo=xyz for rectangular coordinates, or for cylindrical coordinates

0, 0, 0 =x, y, z coordinates of the beginning of your mesh.

iii= locations of coarse mesh points in the x direction if rectangular, or r direction if cylindrical

III= number of fine mesh points that will go between every coarse mesh point listed in iii

jjj= locations of coarse mesh points in the y direction if rectangular, or theta direction if cylindrical

JJJ= number of fine mesh points that will go between every coarse mesh point listed in jjj

kkk= locations of coarse mesh points in the z direction for both rectangular and cylindrical

KKK= number of fine mesh points that will go between every coarse mesh point listed in KKK
